# Effects of message delivery on cross-cultural biosecurity compliance: Insights from experimental simulations

**DOI:** 10.3389/fvets.2022.984945

**Published:** 2022-11-16

**Authors:** Tung-Lin Liu, Scott C. Merrill, Aislinn O'Keefe, Eric M. Clark, Ollin D. Langle-Chimal, Luke Trinity, Trisha R. Shrum, Christopher Koliba, Asim Zia, Timothy L. Sellnow, Deanna D. Sellnow, Julia M. Smith

**Affiliations:** ^1^Social Ecological Gaming and Simulation Lab, University of Vermont, Burlington, VT, United States; ^2^Food Systems Graduate Program, University of Vermont, Burlington, VT, United States; ^3^Gund Institute for Environment, University of Vermont, Burlington, VT, United States; ^4^Department of Plant and Soil Science, University of Vermont, Burlington, VT, United States; ^5^Department of Animal and Veterinary Sciences, University of Vermont, Burlington, VT, United States; ^6^Vermont Complex Systems Center, University of Vermont, Burlington, VT, United States; ^7^Department of Computer Science, University of Victoria, Victoria, BC, Canada; ^8^Department of Community Development and Applied Economics, University of Vermont, Burlington, VT, United States; ^9^Department of Computer Science, University of Vermont, Burlington, VT, United States; ^10^Nicholson School of Communication, University of Central Florida, Orlando, FL, United States

**Keywords:** uncertainty avoidance, loss aversion, risk communication, livestock biosecurity, compliance, cross-cultural communication, experimental simulation, serious games

## Abstract

**Background:**

Effective biosecurity communication of transmission risks and associated protective behaviors can reduce the impacts of infectious diseases in US animal agriculture. Yet, more than 1/5 of animal production workers speak a language other than English at home, and more than 40 percent are less than fluent in English. Communicating with these workers often involves translating into their primary languages. However, communication strategies targeting different cultural groups are not well-understood.

**Aims:**

To identify cross-linguistic risk communication strategies to facilitate compliance, we hypothesized that uncertainty avoidance cultures associated with the languages might affect biosecurity compliance contingent upon two additional covariates: (1) the risk of acquiring an infection and (2) the delivery method of the infection risk.

**Methods:**

We designed an experimental game simulating a line of separation (LOS) biosecurity tactic in a swine production facility, where participants were tasked with completing tasks inside and outside of the facility. Data were collected using games in the two most spoken languages in the US: English (EN) and Spanish (SP). Participants made binary decisions about whether to use the LOS biosecurity tactic based on the risk information provided. Mixed-effect logistic models were used to test the effects of covariates on using the LOS tactic by different language groups.

**Results:**

We found that biosecurity compliance rates of participants who took the experiments in the language associated with high and low uncertainty cultures showed no significant differences. However, there are substantial differences in how risk information is perceived between the two language groups under different infection risks. Specifically, and counterintuitively, SP participants were more risk-averse in gain scenarios but more risk-taking in loss scenarios. These differences are most pronounced in numeric risk messaging, indicating that numbers may not be the best way to communicate risk information regarding biosecurity cross-culturally.

**Conclusions:**

When confronted with situational biosecurity decisions, risk perception and preferences vary by language group. Effective biosecurity communication needs to account for these differences and not assume that direct translation of risk messages will result in comparable compliance.

## 1. Introduction

Compliance with biosecurity practices reduces transmissions and the economic impacts of infectious diseases in animal agriculture. An outbreak of African Swine Fever could cost the US pork industry alone *$*50 billion over 10 years (1). However, biosecurity in animal production involves coordinated human decision-making across the livestock production chain (2, 3). At the farm level, regular biosecurity practices, such as implementing sanitation protocols before entering a production facility, effectively reduce the spread of diseases (4). However, compliance with these practices on farms is inconsistent across regions and countries (5, 6). Even though Workers are familiar with biosecurity practices, they often face constraints such as time to adhere to these practices and complete their day-to-day tasks (7). In some countries, the presence of seasonal farm workers that are less familiar with these practices may also lower the compliance rate and increase the risk of diseases (8).

To reduce the threat of transboundary animal diseases in globalized livestock industries, it is increasingly important to understand cross-cultural influences in the decision-making process for biosecurity compliance. Compliance with on-farm biosecurity practices is influenced by various socio-psychological factors (9, 10). These include the characteristics of disease risk factors and their associated costs to implement or comply with these practices (11), how the risk of diseases is communicated and perceived (12), and heuristics used to inform biosecurity judgment (13–17). Motivation and conditions of such decisions might also vary at tactical (i.e., decision-making at the owner or operator level) and operational (i.e., decision-making at the on-farm worker level) levels (15, 16) and across different populations.

Cross-national assessments of farm-level biosecurity compliance are still emerging (18). Many other studies have examined these differences outside of a biosecurity decision-making context. Such decision-making factors include risk perception and risk preference, and they tend to vary across application domains (19–22). Of the five Hofstede cultural dimensions (23), the uncertainty avoidance index (UAI) that measures countries' tolerance to uncertain and ambiguous situations is most relevant to risk preference and commonly adopted to explain the differences (24, 25). Countries that are intolerant to ambiguity and lack structural situations tend to be more risk-averse in general. In a large-scale 35 country-study, Rieger and colleagues found that countries with high UAIs are more risk-averse to potential gains but more risk-seeking in potential loss (25). Risk aversion when dealing with gains and risk seeking when dealing with losses or “loss aversion” is consistent with prospect theory (26). Between country variance of UAIs can explain the cross-cultural patterns of decisions under risk (27).

**Figure 1:**
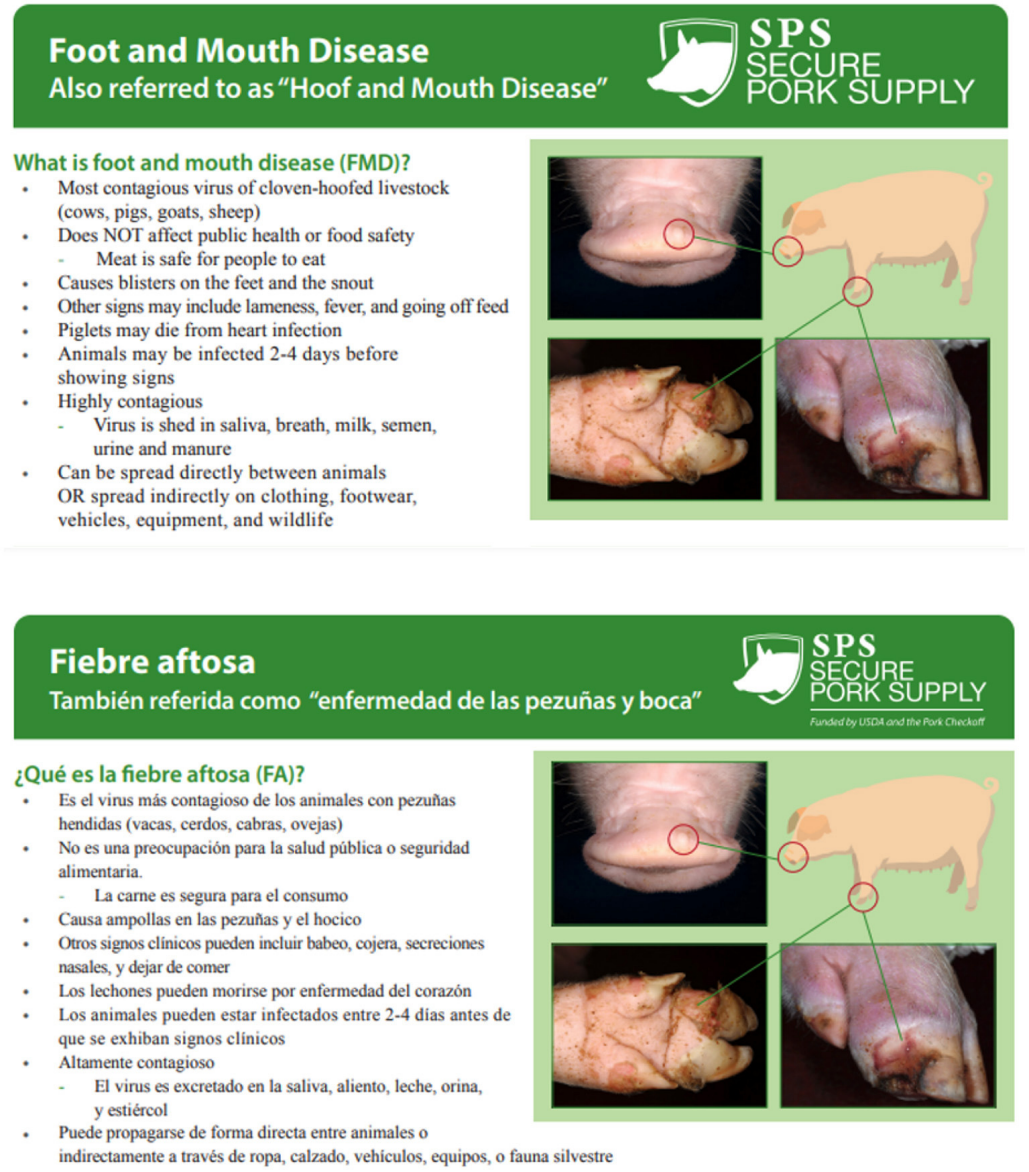
Multi-lingual biosecurity resources concerning foot and mouth disease (credit: Secure Pork Supply).

According to Hofstede (23), the United States has a low uncertainty avoidance index at the national level (UAI: 46). However, several socio-demographic factors can influence aversion to risk at the individual level (28). Here, we note that there are overlaps between the language spoken and socio-demographic factors, especially in the livestock industry. More than 20% of US animal production and processing workers speak a language other than English at home, and more than 40% of that group are less than fluent in English (29). Spanish is the most spoken language after English. Many migrant and seasonal farmworkers are also natives of Spanish-speaking countries (30), with UAIs ranging from 82 (Mexico) to 94 (El Salvador). To disseminate important biosecurity information, multilingual resources are commonly used to communicate disease information and associated risks to non-native or limited English proficient individuals and communities (see Figure 1).

Using the primary language of the intended audience is cited as having a positive effect on increasing trust in science communication (31) and the perceived trustworthiness of risk messages (32). Translating biosecurity guidelines also encourages cooperation and knowledge transfer across language and cultural borders (33, 34). However, translations used in these biosecurity materials could also affect aspects of information communication. Technical terms originating from English publications may not have equivalent translations, cultural connotations, or neither two (33, 35). One study found that Spanish translations of English technical manuals often reflect the linear and direct discourses or “low-context cultures” in the United States but not the digressive or indirect styles in “high-context cultures,” such as Mexico and many Latin American countries (36).

Because language functions as a critical carrier of human culture and co-evolved with the cultural framework (37), processing in different languages can affect risk perception (38), and studies show that using languages associated with low uncertainty avoidance culture may lower the perceived risk in an online environment. (39, 40). For instance, Spaniards' perceived risk is substantially reduced when the information is presented in English, whereas Britons' perceived risk slightly increased when the information is presented in Spanish. Therefore, when both English (associated with low UAI) and Spanish (associated with high UAI) are accessible to bilingual farm workers, communicating in Spanish may have an advantage over English in increasing perceived risk.

Nevertheless, the relationships between culture and language are complex and remain underexplored in the domain of biosecurity. Presenting risk information in languages associated with different cultures not only affects perception, but languages also can shape reasoning (41) and thinking on abstract ideas like time (42) and space (43) that are all critical in risk communication and biosecurity decision-making. Furthermore, contextual factors determine how risk is understood (44). Risk perceptions vary based on a variety of factors, including financial, health, and other domains (45). Therefore, interpreting these cross-cultural differences in risk perception and biosecurity decision-making and identifying culturally sensitive risk translations to overcome these differences is essential.

Computer-based serious games provide an integrated approach to studying the mechanics of decision-making in a controlled environment (46, 47). Merrill et al. used an experimental simulation game of a swine production facility to quantify the situational risk associated with agricultural biosecurity decision-making (16). The research group found several factors influencing disease perception and compliance with the “shower-in and shower-out” biosecurity practice observed in the game. Incrementally increasing infection probability levels and uncertainty associated with risk messages increased the likelihood of compliance. In a later edition of the game (Compliance Game Version 3.0 English), they found evidence supporting linguistic and graphical risk messaging over numeric risk messaging to increase in-game compliance (17).

Our goal was to identify effective cross-cultural risk messaging strategies to promote biosecurity compliance, considering the necessity of translation and moderating the effects of different cultures and languages on risk communication, perception, and biosecurity compliance in the United States. Thus, we deployed the Compliance Game Version 3.0 English to compare with a new sample of participants using a translated Spanish version. We chose Spanish because it is more than 1/5 of animal production and processing workers' primary language and is also the second most spoken language (16.3%) in United States households after English (70.5%) (29).

The findings from previous versions of the games and Version 3.0 and the translated Spanish version formed the basis of our research in (1) the potential differences in biosecurity compliance between socio-linguistic groups and (2) the influence of the use of other primary languages on biosecurity materials and risk messaging on compliance in the United States livestock system.

Specifically, we hypothesize that:

H1: There are significant differences in observed compliance between languages associated with high and low uncertainty avoidance cultures—the languages associated with high uncertainty avoidance cultures have higher perceived risk and thus compliance. Participants in the Spanish version of the game have higher compliance than participants in the English version;
H2: Languages associated with high uncertainty avoidance cultures are more loss averse. Participants in the Spanish version of the game are more risk-averse and compliant in gain scenarios and more risk-taking and non-compliant in loss scenarios;
H3: Compliance increases with the following risk message delivery methods: numerical, linguistic, and graphical (H3.1). The compliance differences between language groups are observed when infection risk messages are delivered using non-linguistic (i.e., numeric) and linguistic means (i.e., linguistic, graphical threat gauge with linguistic phrases) due to translations (H3.2).

Consistent with the findings of Merrill et al. (17), we anticipate that all participants increase their willingness to comply with the “shower-in, shower-out” biosecurity practice as the infection probability and the uncertainty associated with risk messages rise (48).

## 2. Methods

To address the impacts of non-English languages on operational biosecurity compliance challenges faced in the pork industry, we deployed English and Spanish versions of Compliance Game (V3.0) to simulate decisions that farm workers face in day-to-day operations (17). The dependent variable measured in the game is binary decisions to use a “shower-in and shower-out” biosecurity tactic under different experimental parameter combinations, which we call treatments.

### 2.1. Experimental design

**Figure 2:**
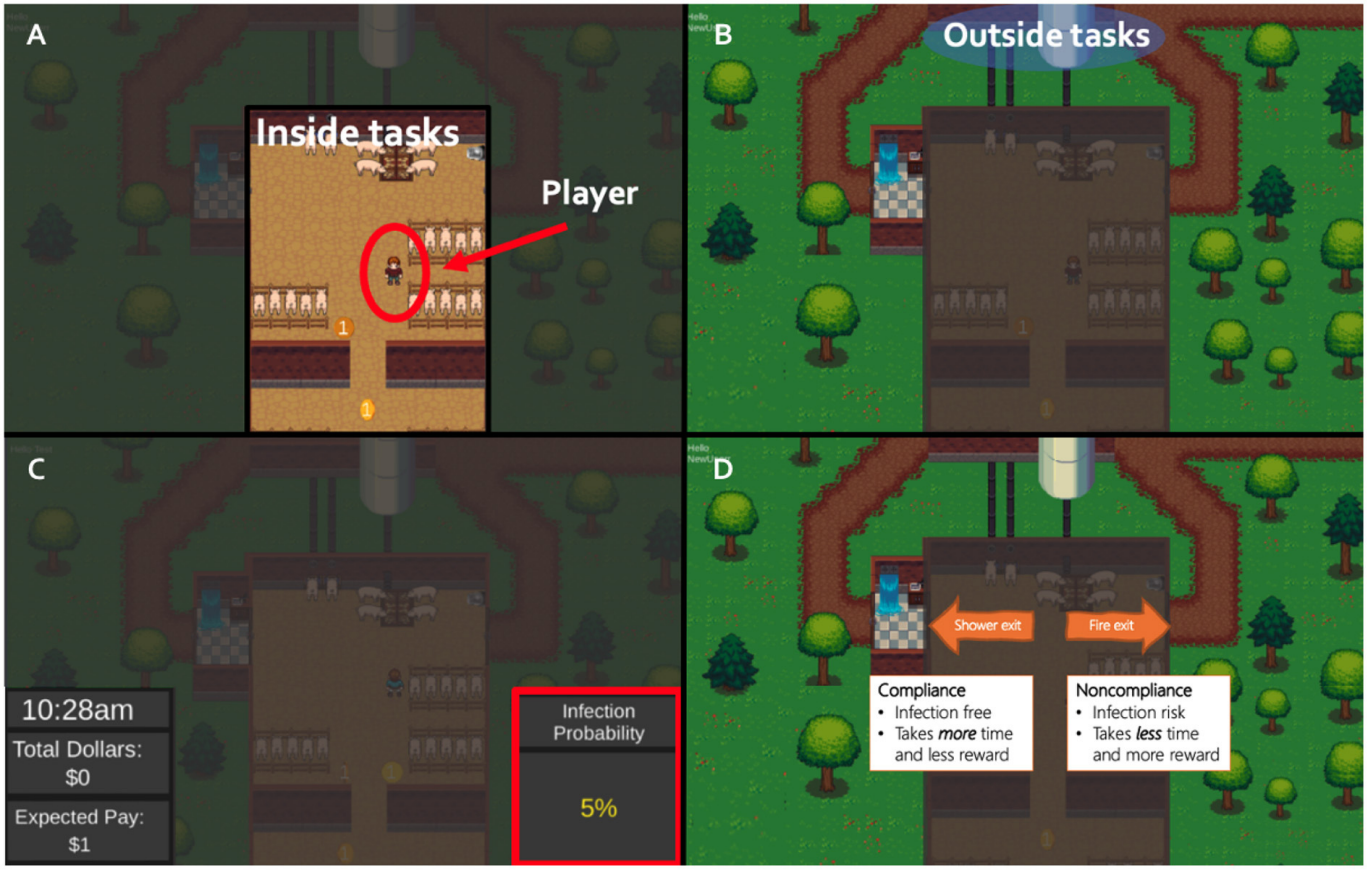
The compliance game and the decision mechanism. **(A)** A player in the inside task area. **(B)** A blue circle indicates the outside task area. **(C)** To go from one area to another, participants have to decide to follow the shower biosecurity practice (left arrow) or break the biosecurity practice to use the emergency exit (right arrow) using the risk information. **(D)** Using the shower exit takes more time and has a lower monetary return than the emergency exit, but it guarantees no infection will occur at the given round.

The game was built using the Unity Development Platform (Unity Technologies, Version 5.6.3) and was hosted online using WebGL (49). The experiment had 24 rounds that varied by the infection risk information provided (i.e., infection risk message treatments; see Table 1). Each participant played the role of a farm worker (Figure 2A). Each round lasted up to 60 s representing one condensed workday from 9 a.m. to 5 p.m. In addition to the 24-treatment rounds, the experiment began with a practice round. Participants earned experimental dollars convertible to U.S. dollars.

**Table 1:** Experiment treatments and the number of round per treatment by participant group.

**Treatment**	**English** **(*n* = 1,305)**	**Spanish** **(*n* = 287)**
Infection risk: 1% (very low)	7,830	1,722
Infection risk: 5% (low)	7,830	1,722
Infection risk: 15% (high)	7,830	1,722
Infection risk: 25% (medium)	7,830	1,722
Diagnosis certainty: “certain”	15,660	3,344
Diagnosis certainty: “uncertain”	15,660	3,344
Message delivery: numeric (1, 5, 15, 25%)	10,440	2,296
Message delivery: linguistic (very low, low, medium, and high)	10,440	2,296
Message delivery: graphical (a threat gauge with arrows pointing to a linguistic phrase)	10,440	2,296

A typical round consisted of four stages:

Stage 1: Risk messages
An infection risk message displayed information on the screen according to the treatment. The same message was displayed throughout the round (Figure 2C);
Stage 2: Task inside the barn
Each round started at 9 a.m. A farm worker avatar appeared inside the barn and was prompted to begin conducting tasks within the facility, represented by spinning coins (Figure 2A). Participants used arrow keys on the computer keyboard to move the avatar to a coin, each worth one experimental dollar. Spinning coins appeared at a rate of one coin every 2 s;
Stage 3: Outdoor task and biosecurity decision
At different times of the day, an outdoor task would appear outside the facility (Figure 2B). The outdoor tasks had a different reward structure than the inside tasks. The reward of the outdoor task started with $30 experimental dollars and decreased by $1 per second. Participants were then asked to choose how to leave the facility to complete the outside tasks. To earn the reward, they would have to decide whether to follow the “the shower-in and shower-out” practice or break the practice to use an emergency exit to reach the outside task (Figure 2D). The “shower-in and shower-out” practice took 5 s to exit and another 5 s to re-enter to resume inside tasks. Thus, complying with the practice resulted in forgoing the 5 s during which the outside task reward was decrementing ($5) and 5 s to resume inside tasks with coins appearing once every 2 s ($2.5), but using the “shower-in and shower-out” practice guaranteed that pigs would not become infected. If participants chose to exit the facility using the emergency door, they would not incur a time penalty. However, using the emergency exit risked an infection in their pigs, with the infection risk varying by treatment. Specifically, infection risk was associated with a probability, and infection occurrence was determined using a random number generator;
Stage 4: End of the round
If infection occurred, the round ended immediately. Otherwise, each round ended at 5 p.m. At the end of each round, the experimental dollars earned and earnings accumulated thus far would be displayed. A $50 penalty would be imposed if an infection occurred each time, as well as loss of any accrued earnings during the round (i.e., money earned from collecting coins within the facility and completing the outside task).

### 2.2. Translation and recruitment

The experimental game and questionnaires were developed in English and translated by researchers into Spanish. Native speakers verified the translations according to the original text. A sample of the U.S. population was recruited using two versions of the games on the online workplace Amazon Mechanical Turk (50–52). For participants taking the non-English version, a language qualification question was included to ensure participants had a working knowledge of the languages. To increase engagement, recruits were informed that their pay would be based on performance during the experiment (53). Institutional Review Board approved practices for an experiment using human participants (University of Vermont IRB # CHRBSS-16-232-IRB).

### 2.3. Experimental treatments and variables

A complete block design was implemented to test factors influencing willingness to comply under risk (Table 1). For each language, we compiled risk messaging using 12 combinations of infection probabilities (1, 5, 15, 25%) and delivery method treatments (Numeric, Linguistic, and Graphical). Note the risks are elevated compared to reality to allow analyzing the changes in infection rate in relation to inadequately using a biosecurity practice.

We posit that the following features could be predictive of the next emerging strain:

1. Numeric: Risk information displayed numerically: 1, 5, 15, or 25%;
2. Linguistic: Risk information displayed linguistically: “Very Low,” “Low,” “Medium,” or “High;”
3. Graphical Threat Gauge: Risk information is illustrated using a threat gauge with an arrow pointing to a linguistic phrase: “Very Low,” “Low,” “Medium,” or “High.”

**Figure 3:**
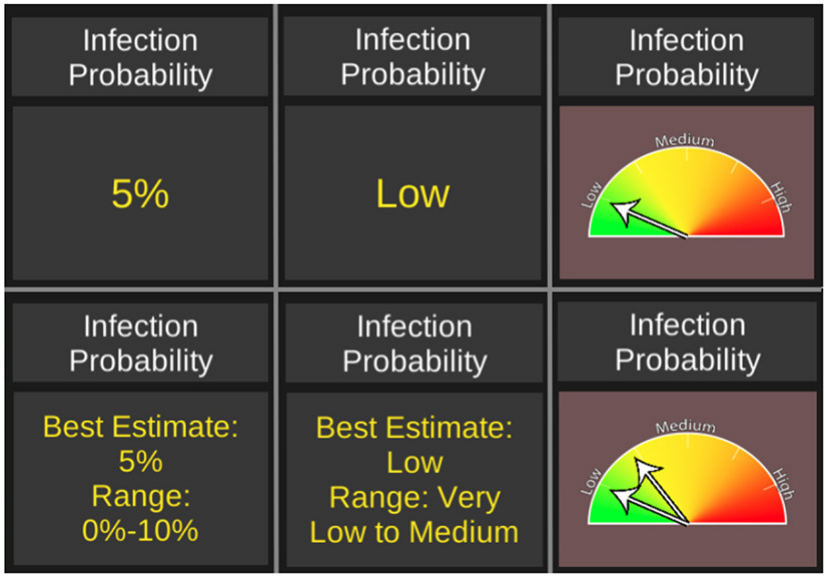
A sample of treatments used in the experiments. Numeric, linguistic, and graphical messaging of 5% (low) infection risk in English **(top)**. Same messaging under uncertainty treatment **(bottom)**.

Each of the 12 combinations listed above was displayed with a certain, absolute risk infection value or as an uncertain risk infection value for 24 treatments. For each risk message treated with uncertainty, a fixed value from above was provided as the best estimate with a range of risk values. For example, a linguistic message with low risk would be shown as “Best estimate: Low; Range: Very Low to Medium (see Figure 3).” Altogether, each participant received 2*12 treatments in a random order in the language given. As indicated, participants utilized the treatment information provided in their respective languages in each round to make a binary biosecurity decision to either exit the facility using the “shower-in, shower-out” biosecurity practice or avoid the opportunity costs associated with biosecurity practice by using the emergency exit (Figure 2C). The mean opportunity cost was estimated at $8.67, considering the additional time spent getting outside and the loss of opportunity to collect coins inside the facility upon early return.

There is an economic trade-off between the two choices. For example, if the infection risk is at 15%, participants chose between the opportunity to gain approximately $8.67 and the risk of a $7.5 expected loss ($50 infection penalty × 0.15 = 7.5). Based on the reward of the tasks and expected penalty of acquiring infection, when the infection risk is at 15 or 25%, participants are in a loss scenario, and the optimal strategy is to be risk averse and comply with the shower practice; however, when the infection risk is low, participants are in a gain scenario, and the optimal approach is to be risk-taking and use the emergency exit.

### 2.4. Analysis

To explain the binary biosecurity decisions of individuals, we analyzed the data using mixed-effect logistic regression models in R statistical programming language (54–56). All candidate models included participants as a random effect and all treatment variables, including the language using sum coding, as fixed effects (H1). Two additional variables were also used: First, to account for within-experiment learning over 24 rounds, the order of play (1–24) was used to explain the trend of compliance over time (57, 58). Second, fear extinction or the number of rounds since the last time infection occurred in the game (59). These two variables informed compliance behaviors in the previous experiments (16, 17).

All the fixed effects form the base of the model (Model 1). Additionally, to test our hypothesis about the difference in risk perception (H2) and effectiveness of message type across groups (H3), we also regressed the binary decisions against all possible combinations of two-way interactions between message delivery methods, infection risk, and language (Models 2–8). Finally, we also tested the three-way interaction of three treatments (Model 9).

Given our sample data, we identified the optimal fit of plausible models (Models 1 to 9) using Akaike's Information Criterion (AIC) (60, 61). The model with the lowest AIC score indicates the most parsimonious candidate model that explains the most variations in dependent variable with the fewest parameterized variables (62). Candidate model selection and detailed results of the model are presented in Supplementary Table 1.

## 3. Results

Biosecurity decisions were obtained from 1,592 qualified participants from Amazon Mechanical Turk. 1,305 participants (81.97%) completed the experimental game in English, and 287 (18.03%) in Spanish. The sample closely aligns with the percentages of languages spoken in the United States (29). Participants each completed 24 treatment combinations in their respective languages, totaling 38,208 binary decisions. Of 26,424 decisions to use the “shower-in, shower-out” practice, participants received an average of $25.57 experimental dollars. Of 11,784 decisions to use the fire exit, participants received an average of $32.57 experimental dollars factoring in the penalty when the animals became infected. Participants completed the experiment with a median time of 23.46 min in English and 24.78 min in Spanish.

Table 2 shows the observed frequency of compliance and 95% Wilson score interval to show the uncertainty we have in the sample estimates. The statistical inferences are generated from the AIC-selected best candidate model (Model 8, see Supplementary Table 1). The mixed effect model included participants as a random effect and all treatment variables as fixed effects, including the two-way interactions between message delivery method (M), infection risk (IR), and language (L). Given our data, including a 3-way interaction of the three variables does not improve the AIC score and risks of losing information by overfitting (Model 9).

**Table 2:** Observed frequency of shower practice by treatment and covariate interaction.

**Infection risk (%)**	**Messaging** **delivery method**	**Language**	**Mean**	**95% lower bound**	**95% upper bound**
25	Graphical	Spanish	0.8031	0.7686	0.8336
25	Graphical	English	0.9169	0.9056	0.9268
25	Linguistic	Spanish	0.8206	0.7871	0.8498
25	Linguistic	English	0.9165	0.9052	0.9265
25	Numeric	Spanish	0.8101	0.7760	0.8401
25	Numeric	English	0.8556	0.8415	0.8685
15	Graphical	Spanish	0.7944	0.7595	0.8255
15	Graphical	English	0.8908	0.8783	0.9022
15	Linguistic	Spanish	0.8084	0.7742	0.8385
15	Linguistic	English	0.8912	0.8787	0.9026
15	Numeric	Spanish	0.7544	0.7175	0.7878
15	Numeric	English	0.7510	0.7340	0.7672
5	Graphical	Spanish	0.6533	0.6135	0.6911
5	Graphical	English	0.6421	0.6236	0.6603
5	Linguistic	Spanish	0.6794	0.6402	0.7163
5	Linguistic	English	0.6368	0.6181	0.6550
5	Numeric	Spanish	0.6794	0.6402	0.7163
5	Numeric	English	0.5257	0.5065	0.5448
1	Graphical	Spanish	0.5819	0.5411	0.6216
1	Graphical	English	0.4701	0.4510	0.4893
1	Linguistic	Spanish	0.5732	0.5324	0.6130
1	Linguistic	English	0.3969	0.3783	0.4158
1	Numeric	Spanish	0.5610	0.5201	0.6010
1	Numeric	English	0.3571	0.3389	0.3757

**Figure 4:**
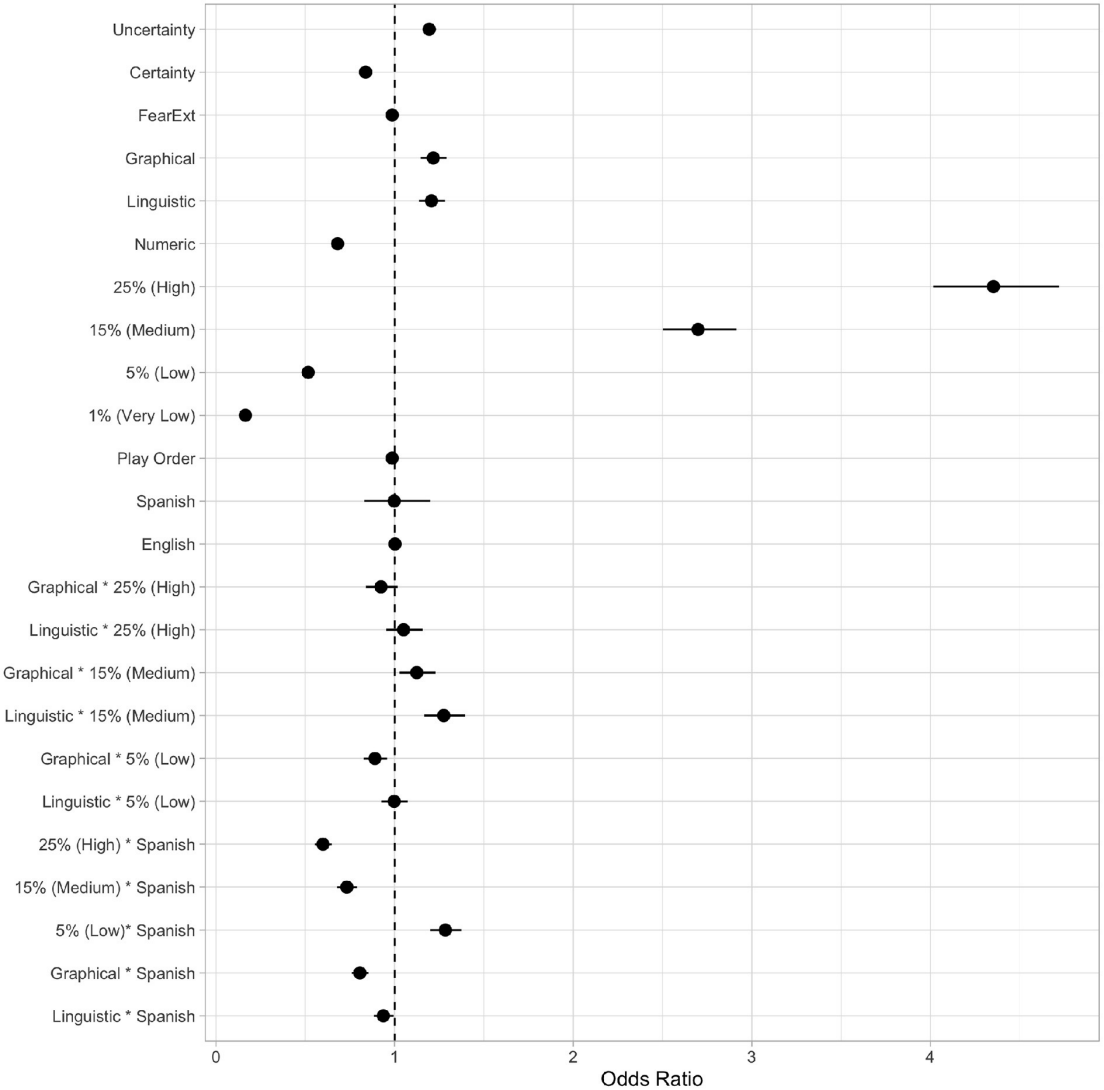
Odds ratio and 95% confidence interval of complying with the shower practice over grand mean. For categorical variables, the reference levels are certainty messaging, numeric messaging, 1% (very low), and English and they are coded as −1 in the model. For categorical variables with more than two levels, it compares each level with the reference level. For example, numeric messaging (−1), linguistic messaging (0), and graphical messaging (1). The asterisk (*) denotes interactions among the variables that it joins.

The logit coefficients are exponentiated into odds ratios describing the odds of using the “shower-in and shower-out” practice instead of the emergency exit. Figure 4 shows the odds ratios and the 95% confidence interval of predictor variables and their interactions used in the model (see Supplementary Table 2 for details). If the odds ratios are greater than 1, participants' odds of using the “shower-in and shower-out” practice are greater than the emergency exit. If the odds ratios are less than 1, the odds of using the emergency exit are greater. Moreover, if the confidence interval includes 1, we cannot rule out the possibility that the odds of compliance and non-compliance are the same. In other words, the variables are not statistically significant.

The logit coefficients of random and fixed effects in the selected model are combined linearly to predict between 0 and 1 to indicate how likely the participants are to use the LOS biosecurity practice under different treatment conditions.

### 3.1. Main effects (H1)

The intercept odds ratio (OR) under sum coding (e.g., −1, 1 for English and Spanish) indicates all categorical treatment effects at the grand mean or zero. It has no specific meaning in our model. Still, the scheme ensures the lower-order effects (as opposed to higher-order effects like interaction terms) can be interpreted as the main effects (overall effect averaged across levels) but not simple effects (effect at one level of the other treatments) when interaction terms are present in the model. For the treatments with more than two levels (i.e., infection risk and message delivery method), the reference levels are 1% (very low) and numeric message delivery.

**Figure 5:**
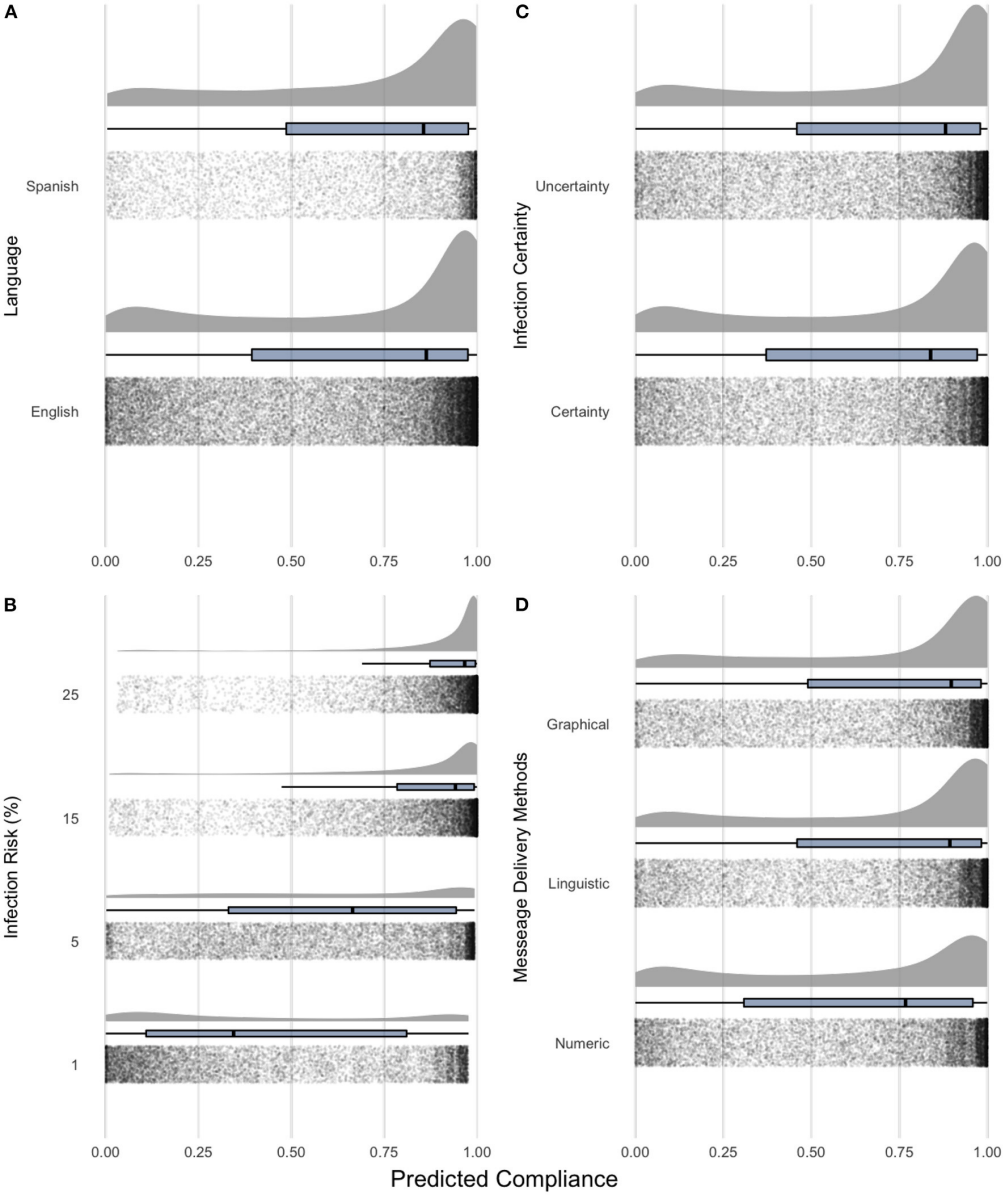
Probability of using the “shower-in and shower-out” practice by the main effects, **(A)** participant groups; **(B)**, infection risk; **(C)** infection certainty; **(D)** message delivery methods. The density and box plots (25th, 50th, and 75th percentiles) are overlaid with model-predicted data values.

We found no compliance differences observed in-game between the two language groups given our sample (see Figure 5A). Participants in Spanish were 0.998 times more likely to use the “shower-in and shower-out” practice than the emergency exit—or, inversely, 1.002 times more likely to use the emergency exit (*p* = 0.981). Contrary to our hypothesis (H1), we did not find evidence suggesting that participants in Spanish (*n* = 287) are more likely to comply with the “shower-in and shower-out” practice than the participants in English (*n* = 1, 305) due to high uncertainty avoidance cultures.

Nevertheless, supporting our previous findings, the use of the “shower-in and shower-out” practices increased when the infection risks rose. Compliance rates rose from 5% (*OR* = 0.516, *p* < 0.0001) to 15% (*OR* = 2.699, *p* < 0.0001), and 25% (*OR* = 4.354, *p* < 0.0001) when referencing to 1% infection risk level (see Figure 5B). Also, consistent with uncertainty research, we found evidence suggesting uncertainty messages increase the compliance rate (*OR* = 1.194, *p* < 0.0001) compared to certainty messages (Figure 5C). Moreover, of the three types of messaging delivery methods, our findings support previous research (17) that suggests linguistic (*OR* = 1.207, *p* < 0.0001) and graphical (*OR* = 1.217, *p* < 0.0001) messages significantly increase the compliance rates compared to numeric messages (Figure 5D).

### 3.2. Covariates (H2-H3)

**Figure 6:**
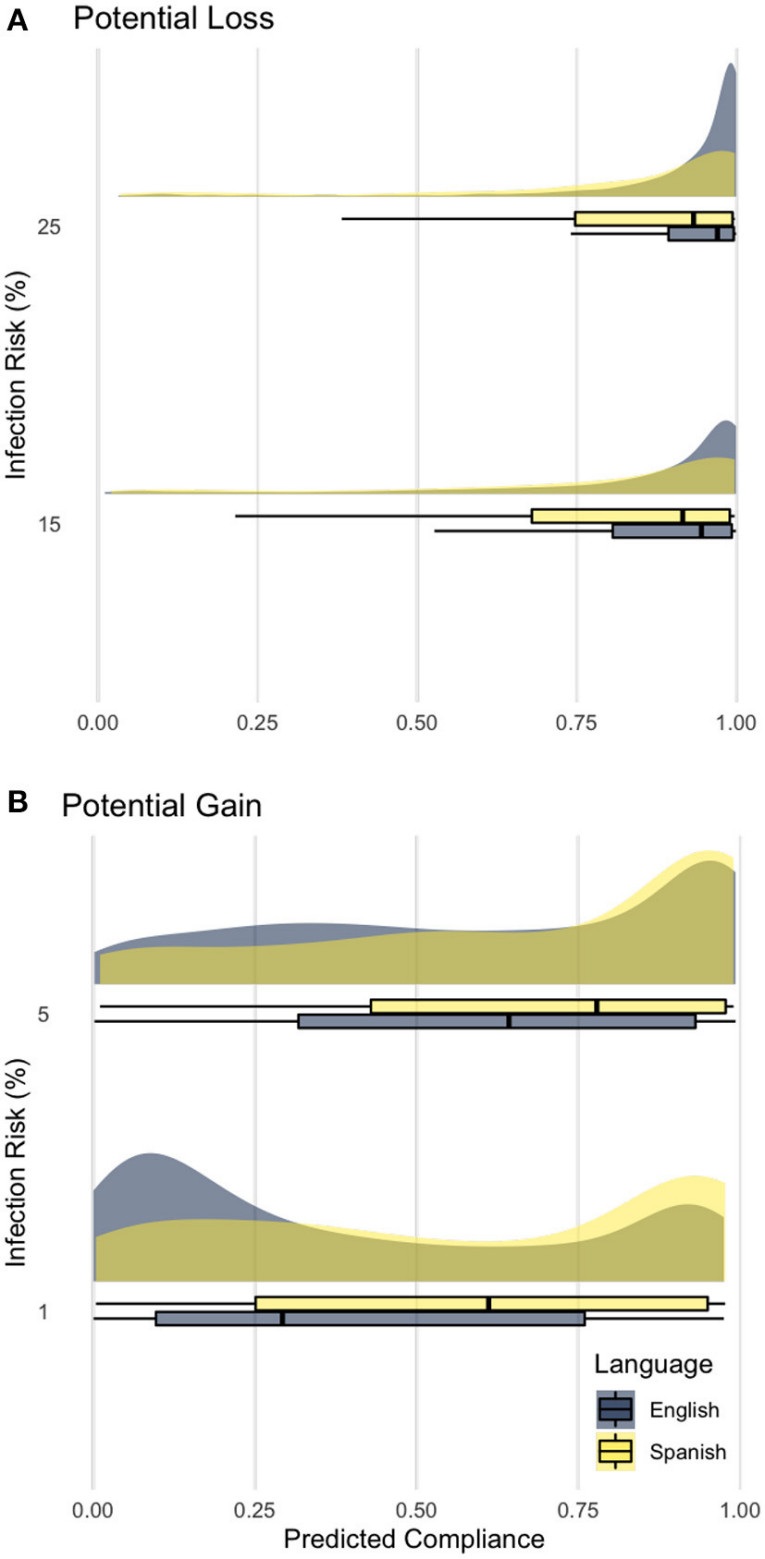
Predicted compliance rate by infection risk and language groups. **(A)** 15, 25% risks yield potential loss without compliance; **(B)** 5, 1% risks yield potential gain with compliance.

Substantial variations found in compliance rates can be explained by the main effects, especially the changes in infection risk. Participants in Spanish are not more likely to comply with “shower-in and shower-out” practices. However, their compliance rates vary significantly by loss/gain scenario associated with infection risks (1 and 5% vs. 15 and 25%) compared to their English counterparts (H2). As hypothesized, Spanish participants are more likely to comply in the gain scenarios at the 5% level (*OR* = 1.284, *p* < 0.0001), but they are less likely to comply in the loss scenarios at the 15% (*OR* = 0.732, *p* < 0.0001) and the 25% (*OR* = 0.599, *p* < 0.0001) levels when compared to the infection risk of 1% reference level. In other words, the change in the effect on the log odds produced by shifting infection risk from gain domain to loss domain at any messaging is different in Spanish group (see Figure 6).

Considering both sub-populations in our sample, our model shows that the increase of compliance rates given delivery methods follow the order of numeric, linguistic, and graphical risk messages (see Figure 5D). However, the difference between linguistic messages (*OR* = 1.207, *p* < 0.0001) and graphical messages (*OR* = 1.217, *p* < 0.0001) is small (H3.1).

**Figure 7:**
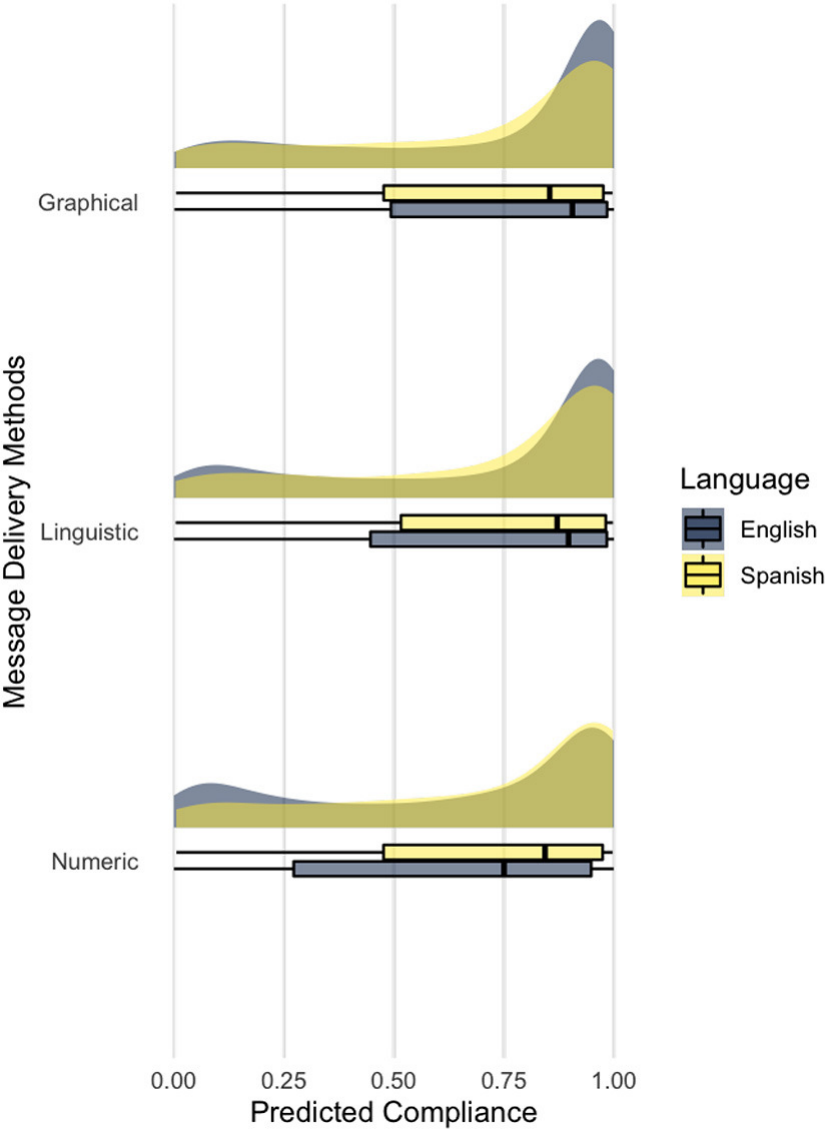
Predicted compliance rate by messaging type and language.

As hypothesized, we observed differences in biosecurity decisions between language groups when risk messages were delivered linguistically (H3.2). Spanish participants are less likely to comply with shower practices with linguistic and graphical messages and English participants with numeric messages. Messages delivered in Spanish phrases have an odds ratio of 0.937 (*p* < 0.05; 1.067 times more likely to use the emergency exit), and the graphical threat gauge with Spanish phrases, 0.805 (*p* < 0.0001; 1.21 times more likely to use the emergency exit). That is to say; we found an interaction between non-linguistic (i.e., numeric) and linguistic (i.e., linguistic and graphical) message delivery methods and their language modalities (see Figure 7). Numeric messages illicit more compliance in Spanish participants and linguistic and graphical messages in English participants. The difference between groups is the smallest in linguistic messaging.

### 3.3. Controlling variables

To control the effects of learning and the temporal distance of the last infection over time on biosecurity compliance, we included the play order (PO) and the rounds since the most recent inflection, fear extinction (FE), in all candidate models. From the best-fitting model (model 8), play order (*OR* = 0.986, *p* < 0.0001), and fear extinction (*OR* = 0.987, *p* < 0.0001), both slightly increase the odds of non-compliance. The inclusions ensure that the effects of other variables and interaction terms in the model are robust and not intermediates through them.

## 4. Discussion

Our experiment aimed to investigate the cross-cultural effects of message delivery on perceived risks and biosecurity compliance. Our work contributes to understanding risk perception and communication to reduce the spread of animal diseases using biosecurity across socio-linguistic groups. We are specifically interested in identifying effective disease risk information delivery for demographic groups in the United States. Here, we sought to understand the relative distribution of risk behaviors in low to high-risk contexts, with specific emphasis on message delivery language.

### 4.1. Uncertainty aversion (H1)

Contrary to our hypothesized association, we found that participants who took the experiments in the language associated with higher uncertainty avoidance cultures were not more likely to comply with “shower-in and shower-out” practices than ones who had taken them in the language associated with lower uncertainty culture (H1). We found no significant differences in shower practice across experimental treatments of different levels between participants in English and Spanish after controlling for other variables. On average, our Spanish sample had a 71.00%, *CI*_95%_[69.91, 72.05] compliance rate followed by English at 68.75%, *CI*_95%_[68.24, 69.27]. The order agrees with our proposed relationship in H1, but we could not rule out the possibility of the difference between English and Spanish due to the limited sample size (see Figure 5A).

These results contradict earlier cross-cultural findings on differences in uncertainty avoidance and risk perception (24). Even though the countries examined were different, we only found that high uncertainty cultures are less risk-taking when accounting for infection risks. One possible explanation is that our hypothesis does not factor in another of Hofstede's cultural dimensions, individualism, which is also linked to risk perceptions (25); for example, the United States and Mexico also have substantial differences (91 vs. 30) in their individualism indexes (23). Also, we modeled Spanish as a fixed effect instead of a random effect due to the limited number of language groups studied (63) and did not consider individual-level differences within the language group. We previously found that the compliance behaviors of simulated workers can socially influence participants within the same culture at the individual levels (10).

Another possible explanation is that Hofstede's uncertainty avoidance is more closely linked to social uncertainty, where people avoid potential social disputes and conflicts, and not environmental uncertainty, where human decisions are not directly related, such as uncertainty in infection risk in our game (64). Environmental uncertainty is more effective than social uncertainty in increasing the willingness to invest in biosecurity protocols (15). Similarly, we found that the uncertainty of risk messaging (i.e., environmental uncertainty) increases the observed shower practice in all our participants (see Figure 5C) but not in uncertainty avoidance cultures (see Figure 5A). Consequently, the between-group variation in compliance signal was most detected considering infection risk levels.

### 4.2. Loss aversion (H2)

As hypothesized, we found that the compliance rates vary significantly by gain and loss domains of infection risk in the languages associated with different uncertainty avoidance cultures (25), suggesting cultural differences in risk perception may be carried out through translation (39, 40). Specifically, the differences between the very low (1%) and low (5%) treatments (i.e., gain domain) and medium (15%) and high (25%) treatments (i.e., loss domain) were most pronounced between the two languages we tested.

As opposed to the group who took the experiment in English, the group that took the experiment in Spanish had much higher compliance rates (linked to risk-averse) when infection risks yield potential gain. Participants in Spanish had much lower compliance rates (linked to risk-seeking) when inflection risks resulted in potential loss. Comparing the changes in compliance across infection risks across two domains, the rates show the mean differences between shower usage diminishes as infection risk climbs (16.4% at 1%; 6.9% at 5%), and the trend reverses when risk levels reach medium and high (−2.6% at 15%; −8.5% at 25%). Therefore, changing how the risk is perceived and increasing compliance by different language groups could pose a significant challenge for mitigating the risks associated with animal diseases with different epidemiological characteristics.

Infection risk has been the main driver of compliance behavior in livestock biosecurity (16, 17). PEDv dramatically impacted U.S. swine production starting in 2013 (65); the outbreak peaked in 2014 with approximately 1.75% of hog facilities infected (15). Therefore, 1% (very low) and 5% (low) are essential risk levels to provide experimental data with empirical relevance. The mean differences can be as high as 16.4% at the same infection risk level. For example, Spanish participants at 1% infection risk were using the “shower-in, shower-out” practice 57.2%, *CI*_95%_[54.85, 59.72] of the time, as contrasted with English participants at 40.8%, *CI*_95%_[39.72, 41.90] usage (16.4% higher compliance amongst Spanish participants).

Thus, degrees of loss aversion, or risk-seeking when in the loss domain, have particular importance to cross-cultural risk communications (66). According to the expected utilities of the game design, the optimal strategies are to use the emergency exit when the risks are low (1 & 5%) and use the shower practice when the stakes are higher (15 & 25%). However, communication information in the language associated with high uncertainty avoidance cultures (e.g., Spanish) reveals a decrease in shower practice rate when risks are high. Communicating risk to non-natives or limited English proficiency using their native languages should not assume their risk preferences are the same as their counterparts.

### 4.3. Message delivery format (H3)

To overcome the differences in risk perception, we hypothesized that the efficacy of infection risk messaging would be lowest using numbers followed by linguistic phrases and graphical indicators such as a threat gauge with corresponding effects on biosecurity compliance for the combined group (H3.1). As hypothesized, our model found that numeric messaging resulted in the lowest shower practice rate, 63.65%, *CI*_95%_[62.81, 64.49]. However, our data were relatively variable and thus did not allow for discernment of a strong signal between linguistic phrases, 71.22%, *CI*_95%_[70.42, 72.00], and graphic threat gauges, 72.61%, *CI*_95%_[71.82, 73.37] yet did support using either of these methods over the use of numbers to describe probabilistic events in the general population.

Nevertheless, when considering differences in the two subsamples, our model showed that participants in Spanish responded to numeric messaging better at 70.12%, *CI*_95%_[68.22, 71.96] than linguistic and more so for graphical messaging type (Figure 7). The order of efficacy is reserved as participants in English responded less favorably to numeric messages at 62.23%, *CI*_95%_[61.30, 63.16]. The reversal of effectiveness between non-linguistic and linguistic messaging may be due to possible translation challenges as both linguistic and graphical messaging involve using linguistic quantifiers (H3.2).

English and Spanish both share recursive numeral systems that use small sets of numbers and generative rules to create number terms like five, fifteen, and twenty-five (67). Numeric values would be more precise tools to communicate risk across these two languages. However, numeric systems are not free of biases. Some numbers occur more frequently (e.g., 1 and 15) in some languages (68). Priming effects of specific numbers (e.g., lucky numbers in different languages) are also observed in economic games (69). The complexity of numeral systems in other languages may explain the observed difference between groups despite the numbers being most likely free from translation errors. Furthermore, number processing demands cognitive loading (67) and familiarity with numeric concepts (70); people's cognitive ability to assess numeric risks to make biosecurity decisions could be impaired under time constraints and biased by heuristic shortcuts (48).

Although linguistic phrases are less precise than numbers, words (e.g., very low, low) can impart a range of probabilities to different individuals (71) and cause people to think in a more categorical way (72). Therefore, these terms and their translations can be used to our advantage to reduce miscommunication and perceptual differences across language groups. Through careful choice of quantifier terms according to the context, translation impacts can be addressed further (73). This may explain why linguistic messaging resulted in the slightest difference between groups. However, for the same reasons, texts are poor at communicating the uncertainty of the events (74). Graphical and visual formats encapsulating data, patterns, and mathematical relationships can be advantageous to those who know to read and interpret them. We expected the combination of colors (i.e., red as danger), spatial orientation (i.e., left to right), and linguistic phrases would have had similar multiplier effects on our English sample (17). However, considering potential differences between participants in English and Spanish, our findings found limited evidence to support the use of graphical messaging over linguistics messaging as it decreases the compliance rates of participants in the Spanish group.

Our graphical messaging design might have missed some conceptualization differences across languages and cultures (75). It is possible that the choice architecture of the threat gauge is foreign to our participants and did not appeal emotionally to them like our English sample did (76). Conflicting evidence, possibly related to translation issues and cultural differences, cautions the use of threat gauge design that combines spatial orientation, color hue, and dial pointed toward the linguistic phrases until further research identifies the effects, including potential miscommunication, of these components to limit the adverse impact on cross-cultural audiences. Target risk messaging for specific language users (e.g., graphical messaging for participants in English and linguistic or numeric messaging for participants in Spanish) is also recommended by the findings, given the current limits of biosecurity translation and cross-cultural risk communication.

### 4.4. Policy implications and recommendations

These findings, at the least, recommend that the translation of biosecurity protocols and tactics should be conveyed in workers' primary languages. Using the primary language is the first step (31, 34). Multi-lingual biosecurity training and communication are needed to reach the sizeable workers with limited English proficiency. At a minimum, risk communicators should also be aware that simple translations of linguistic terms like low, medium, and high could also result in the conveyance of different risk messages, and even the interpretation of numbers or percentages may differ.

Using the primary language of speakers requires not only a multilingual biosecurity glossary to assist in translation and control of vocabularies that are critical to biosecurity (32, 77) but also the understanding of cross-cultural differences that are masked and do not disappear with translations to tailor risk messaging (78)—with the partnership of individuals that speak these languages as their primary languages and multiple gatekeepers (e.g., officials, veterinarians, interpreters, etc.), developing communication strategies that encompass different modalities (e.g., text, voice, etc.) to more accurately describe the risk associated with disease and ensure compliance to reduce risk and increase trust (79).

Granted that farms don't have access to regular threat levels, farms with a higher percentage of primarily Spanish-speaking workers may benefit from higher farm-level compliance but may also be more vulnerable to high-risk events. The communication strategy of disease risk might need to change depending on the disease's infectiousness. For example, some groups might underestimate the threats of highly contagious and deadly pathogens, like the African Swine Fever virus. In contrast, others might minimize the dangers of other less infectious diseases like PEDv.

In some cases, it is best to avoid numbers to communicate these risks as they are not only less understood by English groups but widen the responses by different language users. The use of translated linguistic phrases (e.g., low, medium, etc.) in risk messaging must be attentive to word choices, terms, and metaphors, as equivalents may not be found in the target language. There may be different meanings and interpretations depending on context and people. Graphical threat gauge appeals to cross-cultural audiences are potential areas for further research. To promote compliance across demographic groups in the United States, future work should also continue exploring cross-cultural differences in risk perception and cross-linguistic risk communication strategies to overcome these differences.

The modeling works with a focus on incorporating human factors into disease dynamics at multiple locales can also learn from these significant behavioral differences in compliance across language groups at the operational level or above (65). As more transboundary diseases spread across regions and borders, these nuances can be essential to curb the spread of these diseases and lessen the severity of epidemic waves. These differences may also extend to tactical decisions on biosecurity protocol implementations at the farm level and can potentially interact with the operational compliance given the profile of farm managers and workers in the regions.

### 4.5. Limitation

One limitation of this study: we did not explicitly examine the risk perception difference in language systems to inform the message design by languages by using bilingual speakers and assigning them randomly to English and Spanish versions of the game. Such design can also control unobserved confounders like other cultural values and regional variations amongst the different language groups in the United States. Future work involving the online deployment of games in multiple languages can also benefit from the validated language qualification questions to ensure that participants have the right level of comprehension and are the suitable target group (e.g., bilingual vs. second language users) for the studies.

In our current study, the sample of participants was not recruited based on work experience in swine production. This could lead to biased estimates of the cross-cultural difference between language groups if substantial discrepancies exist between our target population and the online sample. Our sample closely matched the language usage in the United States population. However, evidence of how industry professionals by language group behave differently from the general US population is limited. Clark et al. used a similar experimental methodology and found that agricultural professionals such as business owners and animal health experts did not differ significantly from the online sample in biosecurity investment decisions when under risk (13). Thus, farmers' decision-making processes are confounded by various motivating factors and multiple objectives and are confronted with the complexity of tasks involved, which combined may make discernment of differences between the farmer population and the participants in this study difficult to detect. However, we acknowledge that the absence of context and experience in our sample may affect their risk perception and biosecurity compliance and remains a limitation of the study.

We recognize that the threat of biosecurity is inevitably perceived differently between experimental games and real-world settings. As natural field experiments examining the response of messaging delivery methods by language groups are scarce, detecting the changes in the distribution of behaviors by infection risk can still contribute to the understanding of on-farm behaviors and are also less sensitive to bias (80).

As highlighted previously in this article, we also faced the same challenge in translating the game into different languages. Some concepts in biosecurity may not have an agreed-upon convention in non-English languages. Many terms originate from English-speaking literature. Better translations will ensure the foundation of creating a global culture of biosecurity compliance in animal production. The future of better biosecurity compliance research across global players in the livestock industry also relies heavily upon it.

## 5. Conclusion

Here, we confirm our hypothesis that languages and their associated uncertainty avoidance cultures may shape risk perception and, thus, affect biosecurity compliance. In the United States, participants in Spanish have a mean shower practice rate comparable to participants in English. Still, their compliance behaviors are less adaptive to the rising infection risk levels and the switch from gain domains and loss domains due to loss aversion. To overcome these possible differences in response to different animal diseases and epidemic phases, we recommend linguistic risk messaging to ensure the most consistent compliance with biosecurity practices when communicating to linguistically and culturally diverse audiences.

## Data availability statement

The raw data supporting the conclusions of this article will be made available by the authors, without undue reservation.

## Ethics statement

The studies involving human participants were reviewed and approved by University of Vermont IRB. Written informed consent for participation was not required for this study in accordance with the national legislation and the institutional requirements.

## Author contributions

Data curation was helped by EC and SM. Data analysis and initial manuscript drafts were created by T-LL. Project funding was generated with the help of JS, SM, CK, TS, and AZ. Experiments were conducted by SM and EC. Software development was primarily led by LT and EC. Subsequent manuscript editing and retooling were completed and assisted with the design and conceptualization of the experiment and underlying experimental game by all authors. All authors contributed to the article and approved the submitted version.

## Funding

This work was supported by the USDA National Institute of Food and Agriculture, under award number 2015-69004-23273 and USDA NIFA Award No. 2021-67015-35236.

## Conflict of interest

The authors declare that the research was conducted in the absence of any commercial or financial relationships that could be construed as a potential conflict of interest.

## Publisher's note

All claims expressed in this article are solely those of the authors and do not necessarily represent those of their affiliated organizations, or those of the publisher, the editors and the reviewers. Any product that may be evaluated in this article, or claim that may be made by its manufacturer, is not guaranteed or endorsed by the publisher.

## Author disclaimer

The contents are solely the responsibility of the authors and do not necessarily represent the official views of the USDA or NIFA.
