# Supplementary material for: Effects of message delivery on cross-cultural biosecurity compliance: Insights from experimental simulations
Source: Front Vet Sci. 2022 Nov 16;9:984945. doi: 10.3389/fvets.2022.984945 (PMC9709259; doi:10.3389/fvets.2022.984945)
Supplement: Supplementary file 1 [file Data_Sheet_1.docx]

**Supplementary Material**

**Table S1** Candidate models ordered by AIC value

| **Model** | **IC** | **FE** | **M** | **IR** | **PO** | **L** | **M*IR** | **IR*L** | **L*M** | **M*IR*L** | **AIC** | **ΔAIC** |
| --- | --- | --- | --- | --- | --- | --- | --- | --- | --- | --- | --- | --- |
| 8 | x | x | x | x | x | x | x | x | x |  | 27629.63 | 0.0 |
| 9 | x | x | x | x | x | x | x | x | x | x | 27631.88 | 2.3 |
| 7 | x | x | x | x | x | x | x | x |  |  | 27733.73 | 104.1 |
| 5 | x | x | x | x | x | x |  | x | x |  | 27741.88 | 112.2 |
| 3 | x | x | x | x | x | x |  | x |  |  | 27844.63 | 215.0 |
| 6 | x | x | x | x | x | x | x |  | x |  | 27959.21 | 329.6 |
| 2 | x | x | x | x | x | x | x |  |  |  | 28030.63 | 401.0 |
| 4 | x | x | x | x | x | x |  |  | x |  | 28067.15 | 437.5 |
| 1 | x | x | x | x | x | x |  |  |  |  | 28140.49 | 510.9 |

Random Effect: Participant; Fixed Effects: Infection Certainty (IC); Fear Extinction (FE); Message Delivery Method (M); Infection Risk (IR); Play Order (PO); Language (L). Interaction terms: Message Delivery Method by Infection Probability, is denoted as (M*IP); Infection Probability by Language, is denoted as (LP*L), and so forth.

**Table S2** Results of the selected best fit, mixed-effect logistic regression model (Model 8; see Table S1)

| **Parameter** | **Odds ratio** | **Lower Bound** | **Upper Bound** | **Pr (>\|x\|)** |  |
| --- | --- | --- | --- | --- | --- |
| (Intercept) | 8.667 | 6.771 | 11.094 | 0.000 | **** |
| Infection Uncertainty | 1.194 | 1.156 | 1.232 | 0.000 | **** |
| Fear Extinction | 0.987 | 0.980 | 0.993 | 0.000 | **** |
| Linguistic Message | 1.207 | 1.137 | 1.281 | 0.000 | **** |
| Graphical Message | 1.217 | 1.147 | 1.291 | 0.000 | **** |
| IR @ 5% | 0.516 | 0.482 | 0.553 | 0.000 | **** |
| IR @ 15% | 2.699 | 2.502 | 2.913 | 0.000 | **** |
| IR @ 25% | 4.354 | 4.015 | 4.720 | 0.000 | **** |
| Play Order | 0.986 | 0.982 | 0.991 | 0.000 | **** |
| Spanish | 0.998 | 0.830 | 1.199 | 0.981 |  |
| Linguistic Message by IR @ 5% | 0.998 | 0.926 | 1.074 | 0.949 |  |
| Graphical Message by IR @ 5% | 0.890 | 0.826 | 0.958 | 0.002 | ** |
| Linguistic Message by IR @ 15% | 1.275 | 1.166 | 1.395 | 0.000 | **** |
| Graphical Message by IR @ 15% | 1.124 | 1.028 | 1.229 | 0.010 | * |
| Linguistic Message by IR @ 25% | 1.050 | 0.953 | 1.157 | 0.320 |  |
| Graphical Message by IR @ 25% | 0.924 | 0.839 | 1.018 | 0.108 |  |
| Spanish by IR @ 5% | 1.284 | 1.199 | 1.375 | 0.000 | **** |
| Spanish by IR @ 15% | 0.732 | 0.679 | 0.790 | 0.000 | **** |
| Spanish by IR @ 25% | 0.599 | 0.553 | 0.649 | 0.000 | **** |
| Linguistic Message by Spanish | 0.937 | 0.884 | 0.993 | 0.028 | * |
| Graphical Message by Spanish | 0.805 | 0.760 | 0.853 | 0.000 | **** |
